# Predicted versus observed outcomes in the ALT-FLOW early feasibility study

**DOI:** 10.1093/eschf/xvag101

**Published:** 2026-06-08

**Authors:** Francesco Fioretti, William A Gray, Benjamin Hibbert, JoAnn Lindenfeld, Ryan J Tedford, Firas Zahr, Javed Butler

**Affiliations:** Baylor Scott & White Research Institute, 3434 Live Oak St Ste 501, Dallas, TX 75204, USA; Department of Interventional Cardiology, Main Line Health/Lankenau Heart Institute, Wynnewood, PA, USA; Department of Cardiovascular Medicine, Mayo Clinic, Rochester, MN, USA; CAPITAL Research Group, Division of Cardiology, Department of Medicine, University of Ottawa Heart Institute, Ottawa, ON, Canada; Vanderbilt Heart and Vascular Institute, Vanderbilt University Medical Center, Nashville, TN, USA; Division of Cardiology, Department of Medicine, Medical University of South Carolina, Charleston, SC, USA; Division of Cardiovascular Medicine, Knight Cardiovascular Institute, Oregon Health & Science University, Portland, OR, USA; Baylor Scott & White Research Institute, 3434 Live Oak St Ste 501, Dallas, TX 75204, USA; Department of Medicine, University of Mississippi, 2500 N State St, Jackson, MS 39216, USA

**Keywords:** HFpEF, MAGGIC score, APTURE, Shunt, All-cause mortality

## Abstract

**Background and Aims:**

Whether hemodynamic and symptomatic benefits of the APTURE left atrial-to-coronary sinus shunt translate into clinical outcome benefit in heart failure with left ventricular ejection fraction >40% remains unknown.

**Methods:**

In this post hoc analysis of ALT-FLOW EFS patients undergoing APTURE implantation, Meta-Analysis Global Group in Chronic Heart Failure (MAGGIC) score-predicted 1-year all-cause mortality was compared with observed mortality by Kaplan-Meier analysis of adjudicated events. Heart failure hospitalization (HFH) rates were compared for the 12 months before vs after implantation. Additional analyses included stratification by baseline sodium-glucose cotransporter 2 inhibitor (SGLT2i) use and N-terminal pro-B-type natriuretic peptide (NT-proBNP), recurrent HFH cumulative hazard estimation (Nelson-Aalen), and H2FPEF score calculation.

**Results:**

Among 95 patients (mean age 70.9 ± 8.5 years; 50% women; 93% New York Heart Association Class III; 85.9% with H2FPEF score >3), MAGGIC-predicted 1-year mortality was 13.0%. Observed 1-year survival was significantly higher than predicted (94.7% vs 87.0%; P=0.04), consistent across SGLT2i subgroups (log-rank P=0.59). The proportion of patients with HFH declined from 37.9% pre implantation to 9.6% post implantation (P<0.0001), with event rates falling from 0.61 to 0.21 per patient-year (P<0.001) and a 1-year cumulative hazard of recurrent HFH of 0.19 (95% confidence interval, 0.12-0.31). HFHs were evenly distributed across NT-proBNP strata.

**Conclusions:**

In ALT-FLOW EFS, observed 1-year mortality was lower than MAGGIC-predicted mortality, and post implantation HFH rates were lower than pre implantation rates. These hypothesis-generating findings will require confirmation in the randomized, sham-controlled ALT-FLOW II trial.

## Background

Interatrial shunt devices have not definitely demonstrated clinical benefit in patients with heart failure (HF).^1^ While all other shunts assessing the potential for benefit in HF are interatrial shunts, the APTURE shunt is a left atrial-to-coronary sinus shunt that preserves physiologic flow to the right atrium, potentially leading to improved right ventricular dynamics, and avoids both turbulence in the right atrium due to opposing flow patterns and potential paradoxical embolism. The Early Feasibility Study Of the Edwards APTURE Transcatheter Shunt System (ALT-FLOW EFS) was a single-arm, multicentre trial evaluating the haemodynamic effects, safety, and outcomes of the APTURE left atrial-to-coronary sinus shunt in patients with HF. The 2-year follow-up in patients with left ventricular ejection fraction (LVEF) > 40% showed that the APTURE shunt maintained an acceptable safety profile and patency, with sustained improvements in HF symptoms, quality of life, and functional capacity, regardless of the presence of pulmonary vascular disease, and with no detrimental changes in the right or left ventricular structure and function.^2^

Whether these improvements seen in ALT-FLOW EFS will translate into detectable survival or hospitalization risk benefit remain uncertain as this was a single arm study without a randomized control. The Meta-Analysis Global Group in Chronic Heart Failure (MAGGIC) score is a validated mortality risk model commonly used in patients with HF, which was developed using data from over 39 000 patients across 30 studies, encompassing HF populations with both reduced and preserved LVEF, and incorporates 13 routinely available clinical variables.^3^ External validation studies confirmed the utility of the MAGGIC score in heart failure with preserved ejection fraction (HFpEF), showing significant associations with mortality and morbidity, and comparable performance to other established models, such as the Seattle Heart Failure Model.^4^

## Aims

While there was no randomized control arm in the ALT-FLOW EFS, outcomes of patients were adjudicated for the first-year post implant, providing an opportunity to compare the predicted risk for these patients based on a validated risk prediction scheme with observed outcomes to give an indirect sense about the potential for benefit. In this research letter, we compared the MAGGIC-predicted vs observed 1-year event rates in patients enrolled in the ALT-FLOW EFS. We also compared the 1-year pre- and 1-year post-implantation heart failure hospitalization (HFH) rates.

## Methods

The ALT-FLOW EFS enrolled 95 patients with LVEF > 40% who underwent successful shunt implantation. Meta-Analysis Global Group in Chronic Heart Failure risk scores were calculated for all participants, and predicted 1-year all-cause mortality estimates were derived from the MAGGIC model. Observed 1-year mortality was determined by Kaplan–Meier analysis, with survival curves compared using z-test with log–log transformation. Heart failure hospitalization rates were evaluated in the 12 months preceding and the 12 months following APTURE device implantation. The *P*-value for HFH rate comparison was obtained from a z-test performed after a log–log transformation. All the 1-year events have been adjudicated by the Clinical Events Committee (CEC). The cumulative hazard of recurrent HFH events was estimated using the Nelson–Aalen estimator. Outcomes were stratified by baseline sodium–glucose cotransporter 2 inhibitor (SGLT2i) use and by N-terminal pro-B-type natriuretic peptide (NT-proBNP) above vs below the median. In order to broaden the contextual framework, we also performed an additional analysis on patients with available data using the H2FPEF score, which is a validated diagnostic tool for estimating the probability of HFpEF, incorporating clinical variables, such as obesity, atrial fibrillation, age, and echo parameters.^5^

## Results

### Characteristics of enrolled participants

Among patients enrolled in the study, the mean age was 70.9 ± 8.5 years, 50% of patients were female, and the mean body mass index was 33.7 ± 7.0 kg/m^2^, with a high burden of symptoms (93% participants with New York Heart Association Class III symptoms at baseline). The median NT-proBNP level was 509.0 ng/L [interquartile range (IQR) 137.0–1227.0; *n* = 63]. After 1 year, the median NT-proBNP was 418.0 ng/L (IQR 193.0–1035.0; *n* = 65). The mean resting pulmonary artery wedge pressure and systolic pulmonary artery pressure were 20.1 ± 8.2 mmHg and 44.2 ± 15.6 mmHg respectively, increasing to 35.0 ± 8.5 mmHg and 71.3 ± 17.8 mmHg at 20 W exercise. Among 92 HFpEF patients, the mean H2FPEF score was 5.8 (SD 2.2), with a median of 6.0 (IQR 4.0–8.0), and the majority of patients (85.9%) had a score greater than 3, consistent with a high probability of HFpEF in this cohort.

### Predicted vs observed outcomes

**Figure 1:**
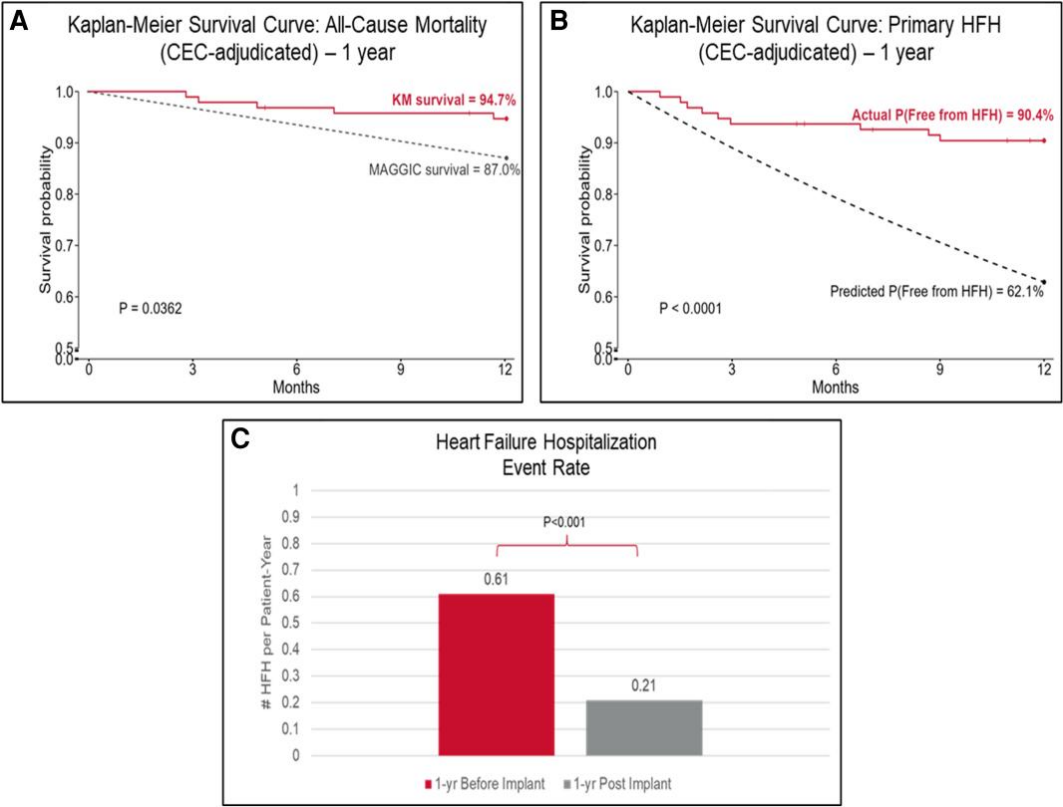
Observed vs predicted mortality based on CEC-adjudicated events and heart failure hospitalization at 1 year. (*A*) Kaplan–Meier curve for actual and predicted survival rates through 1 year; (*B*) Kaplan–Meier curve for actual and predicted rates of patients free from HFH through 1 year; (*C*) HFH event rates 1 year before and 1 year after APTURE device implantation. Analyses included patients with LVEF > 40%, who received APTURE implantation, and enrolled from both the United States and Canada cohorts. CEC, Clinical Events Committee; HFH, heart failure hospitalizations; KM, Kaplan–Meier; MAGGIC, Meta-Analysis Global Group in Chronic Heart Failure; LVEF, left ventricular ejection fraction

All analyses were conducted over a 1-year follow-up period. Over a median follow-up of 148 days, five deaths for any cause occurred among 95 patients by 1 year, including three cardiovascular and two non-cardiovascular deaths, with the majority of the cohort remaining at risk throughout the observation period (*n* = 88 at 12 months), reflecting the overall low mortality rate in this population. A total of 19 CEC-adjudicated HFH events were observed among nine patients at 1 year, corresponding to an average of 2.1 events per patient. When evaluating mortality risk, two patients were censored due to loss to follow-up. When evaluating HFH outcomes, five patients were censored, including two due to loss to follow-up and three due to death. The MAGGIC-predicted 1-year survival for the cohort was 87.0%, corresponding to a predicted mortality rate of 13.0%. In comparison, the observed survival was significantly higher at 94.7% (*P* = .04) (*Figure 1A*). The proportion of patients with a HFH event 12 months prior to shunt implantation was 37.9%, decreasing 12 months following implantation to 9.6% (*P* < .0001) (*Figure 1B*); this equalled to .61 vs .21 HFH events per patient-years, respectively (*P* < .001) (*Figure 1C*).

The Nelson–Aalen estimator was performed to quantify the cumulative hazard of recurrent HFH over time. The cumulative hazard rates for HFH were .19 ± .05, (95% confidence interval, .12–.31) at 1 year per CEC adjudication.

### Stratified analyses by baseline sodium–glucose cotransporter 2 inhibitor use and N-terminal pro-B-type natriuretic peptide

Patients receiving SGLT2i at baseline (*n* = 28) demonstrated numerically lower rates of all-cause mortality and HFH at 12 months compared with those not on SGLT2i (*n* = 67). However, these differences did not reach statistical significance (log-rank *P* = .59 for all-cause mortality; log-rank *P* = .62 for HFH). Interpretation of these findings is limited by the small number of events in both groups, and no definitive conclusions regarding the impact of baseline SGLT2i use on these outcomes can be drawn from this analysis.

We also examined HFH events stratified by baseline NT-proBNP using the median value (509 pg/mL) as the cut-off. Among the 13 CEC-adjudicated HFH events with available baseline NT-proBNP measurements, events were evenly distributed between groups, with seven occurring in patients with NT-proBNP ≤ 509 pg/mL and six occurring in those >509 pg/mL. This finding suggests that, in this cohort, HFH events did not preferentially cluster within higher baseline natriuretic peptide phenotypes.

## Discussion and conclusions

In this symptomatic HFpEF cohort, implantation of the APTURE device was associated with a significantly lower observed 1-year mortality than predicted by established MAGGIC risk modelling. There was also a significantly lower risk of HFH 12 months after device implantation compared with the 12 months preceding implantation. These findings are consistent with previous results from the ALT-FLOW EFS, which reported that patients with HFpEF or mildly reduced LVEF who received the APTURE left atrial-to-coronary sinus shunt had an acceptable safety profile and sustained improvements in haemodynamics and patient-centred outcomes over 1 and 2 years, with all shunts remaining patent and no adverse changes in right heart function.^2^ The divergence between predicted and observed survival rates and between 12-month pre- and post-implantation HFH rates in ALT-FLOW EFS may reflect meaningful physiological impact from left atrial decompression through a novel device, which diverts the blood flow from the left to the right atrium through the coronary sinus, thus preserving the interatrial shunt and the natural right heart vortex flow, which is crucial for efficient filling. However, it is important to note that this was a single-arm, non-randomized feasibility study, with a modest sample size. The observed reduction in HFH rates should be interpreted with caution. In the absence of a concurrent control arm, it is not possible to exclude several alternative explanations for this finding. Regression to the mean is a particularly relevant concern given that 37.9% of patients experienced a HFH in the year preceding enrolment, a period of heightened clinical instability that may have naturally resolved over time regardless of the intervention. Additionally, the intensified monitoring and medical management related to participating in a clinical trial (trial effect) and non-specific benefits of undergoing a procedure (placebo effect) may have contributed independently to the observed reduction in HFH.^1^ These findings should therefore be considered hypothesis generating. A further limitation is the absence of data on medical therapy optimization during follow-up. Since the MAGGIC score was derived before SGLT2i became standard of care, any initiation of these drugs during the trial could have independently improved mortality and HFH rates, making it hard to attribute the observed benefit solely to the device. Although in our cohort the uptake of newer therapies was still relatively modest (e.g. only 25% patients on SGLT2i) due to the timeframe of trial enrolment prior to the guidelines’ update, data on changes in medical therapy during follow-up were not systematically collected. Furthermore, the ALT-FLOW EFS exclusion criteria, which are standard in device feasibility studies, introduce an important selection bias. In particular, the enrolled population is highly selected and does not fully represent the general HF population. Accordingly, the favourable comparison between observed and predicted survival should not be interpreted as proof of a device-mediated mortality benefit but rather as a hypothesis-generating signal. This comparison is further limited by the absence of a concurrent randomized control group. Confirmation of these findings in the ongoing sham-controlled randomized ALT-FLOW II (NCT05686317) trial will be essential to determine whether these findings represent a reproducible treatment effect.

In conclusion, in participants with HF and LVEF > 40% enrolled in the ALT-FLOW EFS, the observed 1-year all-cause mortality was significantly lower than the expected mortality based on the MAGGIC risk modelling. When compared to the 12 months preceding device implantation, a significant reduction in HFH rates in the following 12 months was also shown. These findings suggest a potential clinical benefit. The randomized sham-controlled ALT-FLOW II trial will be essential to clarify whether APTURE implantation in patients with HFpEF translates into improvements in hard clinical endpoints in addition to patient reported outcomes.

## Declarations

### Data Availability

The data that support the findings of this study are available from the corresponding author upon reasonable request and subject to applicable regulatory and privacy restrictions.
